# Evaluation of* Mycobacterium tuberculosis* specific antigen-stimulated CD27^−^CD38^+^IFN-γ^+^CD4^+^ T cells for discrimination of active tuberculosis

**DOI:** 10.1186/s12879-022-07895-1

**Published:** 2022-12-01

**Authors:** Yong Fang, Na Wang, Liang Tang, Xiao-Jun Yang, Yuan Tang, Lin Li, Wen-Fei Wu, Bo Su, Wei Sha

**Affiliations:** 1 Clinic and Research Center for Tuberculosis, Shanghai Key Laboratory of Tuberculosis, Shanghai Pulmonary Hospital, School of Medicine, Tongji University, Shanghai, 200433 China; 2 Central Laboratory, Shanghai Pulmonary Hospital, School of Medicine, Tongji University, Shanghai, 200433 China; 3 Department of pharmacy, Shanghai Pulmonary Hospital, School of Medicine, Tongji University, Shanghai, 200433 China

**Keywords:** Tuberculosis, Diagnosis, Biomarker, Flow cytometry, CD27, CD38, IFN^−^γ

## Abstract

**Background:**

Active tuberculosis (ATB) originates from primary *Mycobacterium tuberculosis* (MTB) infection or reactivation of latent tuberculosis. Besides bacteriological examination, MTB-reactive immunocytes detection can be an alternative testing for discrimination of active tuberculosis. The purpose of this study is to investigate the accuracy of peripheral blood CD27^−^CD38^+^IFN-γ^+^CD4^+^T cells in ATB diagnosis.

**Methods:**

This prospective diagnostic accuracy study was conducted at Shanghai Pulmonary Hospital between January 2019 and December 2021. Patients with ATB, non-tuberculosis mycobacterium infection (NTM), latent tuberculosis infection (LTBI), other respiratory diseases (OD), and healthy individuals (HC) were enrolled. The accuracy of CD27^−^CD38^+^IFN-γ^+^CD4^+^/CD4^+^ and other phenotypic markers for ATB diagnosis was assessed.

**Results:**

A total of 376 patients (237 ATB, 38 LTBI, 8 NTM, 50 OD, and 43 HC) were enrolled. The ratios of CD4^+^IFN-γ^+^CD27^−^ and CD4^+^IFN-γ^+^CD27^−^CD38^+^ profiles in CD4^+^IFN^−^γ^+^ cells and the ratios of CD4^+^IFN-γ^+^CD38^+^, CD4^+^IFN-γ^+^CD27^−^, and CD4^+^IFN-γ^+^CD38^+^CD27^−^ profiles in CD4^+^ cells in the ATB group were significantly higher than in the other groups. The area under the curve (AUC) of CD27^−^CD38^+^IFN-γ^+^CD4^+^/CD4^+^ for the diagnosis of ATB was the highest, with a value of 0.890. With the optimal cutoff value of 1.34 × 10^–4^, the sensitivity and specificity of CD27^−^CD38^+^IFN-γ^+^CD4^+^/CD4^+^ for ATB diagnosis was 0.869 and 0.849, respectively.

**Conclusion:**

CD27^−^CD38^+^IFN-γ^+^CD4^+^/CD4^+^ might be a potential biomarker for active tuberculosis diagnosis.

## Introduction

Tuberculosis (TB) is the ninth leading cause of death in the world, ahead of HIV/AIDS [1]. Despite great advances in TB diagnosis and therapy, the world still faces a huge burden of TB. There are about 2 billion people infected with *Mycobacterium tuberculosis* (MTB) worldwide [1, 2], and China is one of the 30 countries with the highest burden of TB and latent TB infection (LTBI) identified by the World Health Organization (WHO) [3]. It was recently estimated that China had the highest LTBI burden in the world, with approximately 350 million persons living with the infection [4, 5].

The rapid and accurate diagnosis of MTB infection is crucial for TB control and prevention, which is also important for prompt initiation of anti-TB treatment. Conventional diagnostic methods are time-consuming (e.g., TB culture) or have low specificity [e.g., the tuberculin skin test (TST)] [6]. Although Xpert MTB/RIF overcomes the limitations of traditional bacteriological detection methods, which are time-consuming and prone to contamination, the DNA of the present dead or killed bacilli can still be detected months or even years after treatment. Studies have shown [7–9] that Xpert MTB/RIF can detect the DNA of non-viable and incomplete bacteria. For patients with a history of pulmonary tuberculosis, 14.3% of Xpert MTB/RIF positive patients were culture negative and potentially false-positive [7]. In addition, some methods cannot distinguish active TB (ATB) from LTBI [e.g., interferon-γ release assays (IGRAs)] [10]. It is challenging to differentiate ATB from LTBI and other respiratory diseases, and misdiagnosis often occurs [11, 12]. Extra-pulmonary TB (EPTB) accounts for a large proportion of TB [13, 14]. Since the results of etiology and pathology are difficult to obtain for tuberculous serous effusion (such as tuberculous pleuritis, tuberculous peritonitis, tuberculous pericarditis, tuberculous meningitis, etc.), the means of diagnosis and differential diagnosis are limited. However, these molecular diagnosis have limitation in sensitivity, especially for children or extrapulmonary tuberculosis patients, because of the unavailability of representative specimens.

The cellular immune response mediated by T lymphocytes plays a key role in TB infection and pathogenesis [15, 16]. The surface molecules of the T cells are the molecular basis for the mutual recognition and interaction between T cells and other cells and molecules [17, 18]. CD27 is a costimulatory receptor expressed on the surface of CD4^+^ T cells [19]. Early-differentiated CD27^+^ memory CD4 T cells are thought to recirculate primarily in secondary lymphoid organs, while late-differentiated CD27^−^ memory T cells exhibit additional effector functions [20] and preferentially migrate to peripheral sites of inflammation, such as the lungs during ATB [21]. CD38 is a transmembrane glycoprotein expressed on many types of immune cells. It can catalyze the degradation of nicotinamide adenine dinucleotide (NAD) and its precursors that are necessary for bacterial growth [22, 23]. A study showed that CD38 inhibits the metabolism of pathogens by degrading NAD and its precursors in activated immune cells, thus limiting the development or progression of infection [24].

Recent studies showed that compared with LTBI, CD27^−^ and CD38^+^ are mainly expressed in ATB and could be used as markers to distinguish the two diseases [25–27], but there are few studies on the combined CD27^−^ and CD38^+^ profile. Therefore, this study aimed to detect the co-expression of CD27 and CD38 molecules in CD4^+^ and CD4^+^IFN^−^γ^+^ T cells and investigate the accuracy of CD27^−^CD38^+^IFN-γ^+^CD4^+^/CD4^+^ and other MTB-specific phenotypic markers for ATB diagnosis.

## Methods

### Study design and population

This prospective diagnostic study was conducted in Shanghai Pulmonary Hospital, School of Medicine of Tongji University, between January 2019 and December 2021. The inclusion criteria were (1) aged 18–70 years, (2) healthy volunteers (HC) or patients with ATB, LTBI, non-tuberculosis mycobacterium infection (NTM), or other respiratory diseases (OD). The exclusion criteria were (1) primary or secondary immunodeficiency, including HIV, long-term steroid use, or co-existing autoimmune diseases, (2) diabetes or viral hepatitis, (3) history of anti-tuberculosis treatments. This study complied with the Declaration of Helsinki and was approved by the Ethics Committee of Shanghai Pulmonary Hospital. All participants signed the informed consent forms.

ATB (including active pulmonary TB (PTB) and active EPTB) was diagnosed according to (i) no previous history of TB, (ii) positive sputum, bronchoalveolar lavage fluid (BALF), or bacteriological tests, including positive acid-fast staining, positive MTB culture (Bectec960), or positive Xpert MTB/RIF test, and (iii) no other lung diseases [28–30]. LTBI was defined as household contacts of newly diagnosed TB patients with positive IGRA, with no evidence of clinically manifest active TB [31]. OD was defined as patients with any other respiratory diseases except TB, and with negative IGRA results. Healthy volunteers were those had negative IGRA results and no history of TB exposure. Sputum smear-positive patients with negative sputum MTB culture results were diagnosed as non-tuberculous mycobacteria (NTM).

### Data collection

Demographic information including gender and age of all participants was recorded. For each participant, 0.5 mL whole blood was collected in heparin anticoagulant tubes. Acid fast and Xpert MTB/RIF were used for ATB screening. The results of MTB culture and IGRA were recorded.

### ESAT6/CFP10 stimulation and flow cytometry

Within 4 h of blood collection, 0.5 mL whole blood was separately added into sample tubes containing 10 μg/mL ESAT6/CFP10 polypeptide antigens in total (Beckman Coulter, Brea, CA, USA) with Brefeldin A (10 μg/mL, Biolegend, San Diego, CA, USA), and into negative control tubes without ESAT6/CFP10 polypeptide antigens incubated. After 16 h of incubation at 37 °C with 5% CO_2_, 2.5 mL of erythrocyte lysis solution and fixative solution (3% diethylene glycol, 2% formaldehyde, and 0.75% methanol) were added and incubated for 10 min. The cells were centrifuged at 600×*g* for 5 min. The pellet was resuspended in 375 µL Perfix-NC permeabilization solution (Beckman Coulter). Then, 5 μL of IFN-γ-FITC, CD4-PE, CD3-ECD, CD27-PE-CY5.5, and CD38-PE-CY7 were added and incubated for 45 min in the dark. Isotype antibodies were used for FCM gating protocol. The cells were washed with phosphate-buffered saline (PBS) once. After adding 300 μL PBS, a Beckman Coulter DxFLEX flow cytometer was used for rapid collection (60 μL/min). The collection stop condition was set to 100,000 CD4^+^ cells or 300 s of running time. FlowJo V10 (Treestar, Ashland, OR) was used for data analysis. Gating strategy: CD4^+^ T cells were identified from the double positive region of CD3 vs CD4 two-parameter density blots after gating lymphocytes, and then CD4^+^IFN-γ^+^ T cells were delineated for CD27 vs CD38 density blots. Gating process was made by technicians blind of the clinical diagnosis. The ratio of CD4^+^IFN-γ^+^CD38^+^/CD4^+^IFN-γ^+^, CD4^+^IFN-γ^+^CD27^−^/CD4^+^IFN-γ^+^, CD4^+^IFN-γ^+^CD38^+^CD27^−^/CD4^+^IFN-γ^+^, CD4^+^IFN-γ^+^CD38^+^CD27^−^/CD4^+^, CD4^+^IFN-γ^+^CD38^+^/CD4^+^, CD4^+^IFN-γ^+^CD27^−^/CD4^+^ was calculated. Any samples with CD4^+^IFNγ^+^/CD4^+^ greater than 0.02% in negative control were regarded as unqualified samples or measurements, and excluded from the subsequent analysis.

### Statistical analysis

Statistical analyses were performed using SPSS 18 (SPSS, Armonk, NY, USA) and Graph Pad Prism 6 (GraphPad Software Inc., San Diego, CA, USA). Continuous variables are described as means ± standard deviation (SD), and categorical variables as n (%). The Kruskal–Wallis test was used for comparison of independent samples between multiple groups, Dunn’s test was used for pairwise comparison between multiple groups, and the Mann–Whitney U-test was used for comparison of independent samples between two groups. Receiver operating characteristic (ROC) analysis was used to assess the diagnostic performance of each biomarker. The areas under the curves (AUCs) were compared by the DeLong test. The optimal cutoff value of variables was identified according to the maximum Youden index. Two-sided P-values < 0.05 were considered statistically significant.

## Results

**Fig. 1:**
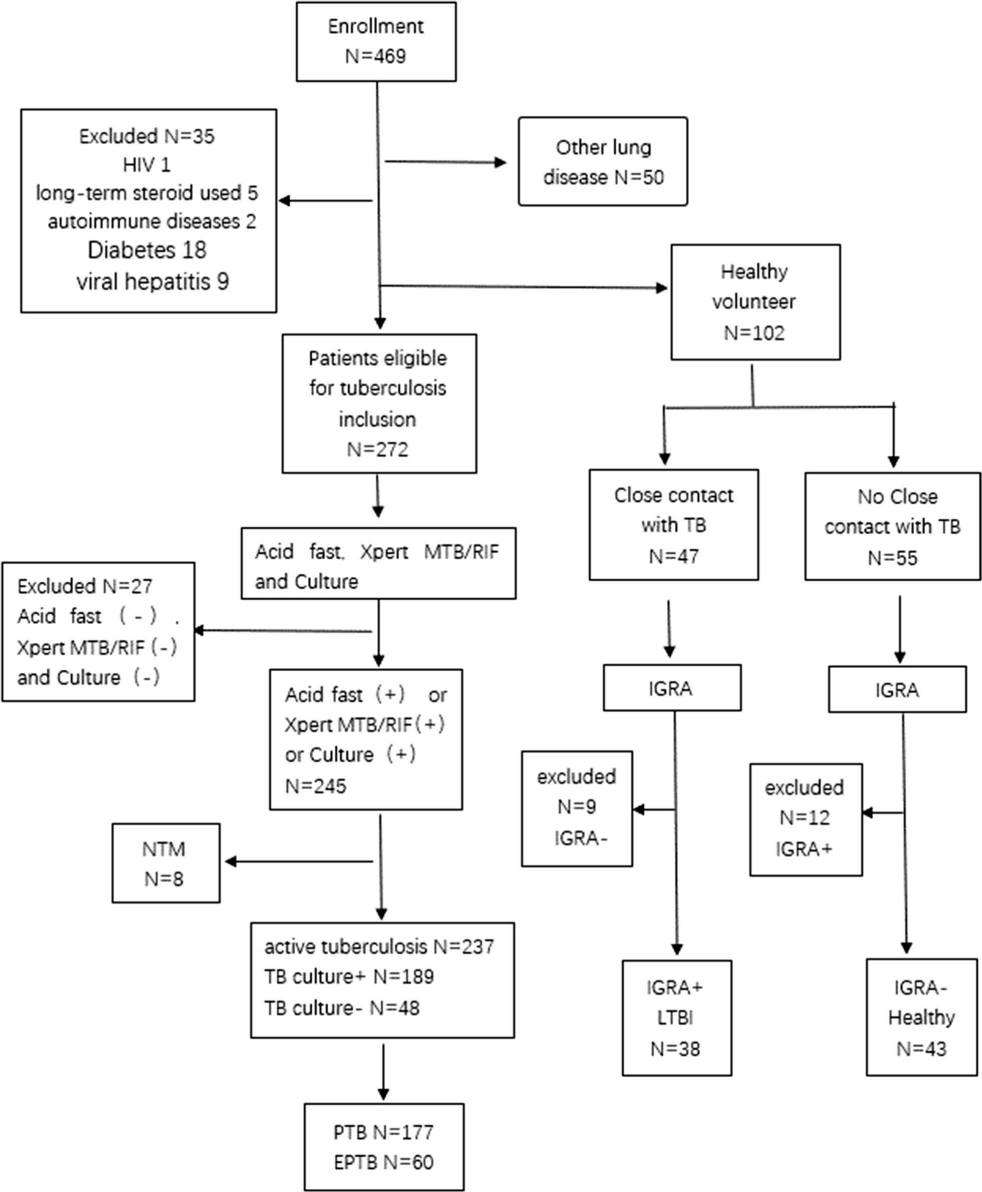
Participant enrollment flowchart. *TB* tuberculosis, *ATB* active tuberculosis, *LTBI* latent tuberculosis infection, *NTM* non-tuberculous mycobacterium pulmonary disease, *OD* other respiratory diseases, *HC* healthy control, *PTB* pulmonary tuberculosis, *EPTB* extra-pulmonary tuberculosis, *IGRA* interferon-γ release assay

A total of 376 participants, including 216 males and 160 females with an average age of 41.2 ± 16.9 years were enrolled (Table 1). There were 237 cases of ATB (177 cases of PTB and 60 cases of EPTB), 38 cases of LTBI, 8 cases of NTM, 50 cases with OD, and 43 HC (Fig. 1).

**Table 1:** Characteristics of the participants

	ATB (n = 237)	LTBI (n = 38)	NTM (n = 8)	OD (n = 50)	HC (n = 43)	P
Male (n, %)	155 (65.4%)	15 (39.5%)	5 (62.5%)	25 (50.0%)	16 (37.2%)	P < 0.001
Age (year)	38.0 ± 16.4	44.6 ± 13.9	48.8 ± 11.0	56.3 ± 16.5	36.5 ± 12.8	P < 0.001
EPTB (n, %)	60 (25.3%)	–	–	–	–	
Acid fast						
N/A (n, %)	0	38 (100%)	0	0	43 (100%)	
Negative (n, %)	144 (60.8%)	0	0	50 (100%)	0	
Positive (n, %)	93 (39.2%)	0	8 (100%)	0	0	
TB culture						
N/A (n, %)	0	38 (100%)	0	3 (6.0%)	43 (100%)	
Negative (n, %)	48 (20.3%)		8 (100%)	47 (94.0%)	0	
Positive (n, %)	189 (79.7%)	0	0	0	0	
Xpert MTB/RIF						
N/A (n, %)	93 (39.2%)	38 (100%)	0	50 (100%)	43 (100%)	
Negative (n, %)	27 (11.4%)	0	8 (100%)	0	0	
Positive (n, %)	117 (49.4%)	0	0	0	0	
IGRA						
N/A (n, %)	21 (8.9%)	0	1 (12.5%)	3 (6.0%)	0	
Indeterminate (n, %)	1 (0.4%)	0	0	0	0	
Negative (n, %)	10 (4.2%)	0	4 (50.0%)	47 (94.0%)	43 (100%)	
Positive (n, %)	205 (86.5%)	38 (100%)	3 (37.5%)	0	0	

*ATB* active tuberculosis, *EPTB* extrapulmonary tuberculosis, *LTBI* latent tuberculosis infection, *NTM* non-tuberculous mycobacterium pulmonary disease, *HC* healthy control, *OD* other respiratory diseases, *N/A* not available

**Fig. 2:**
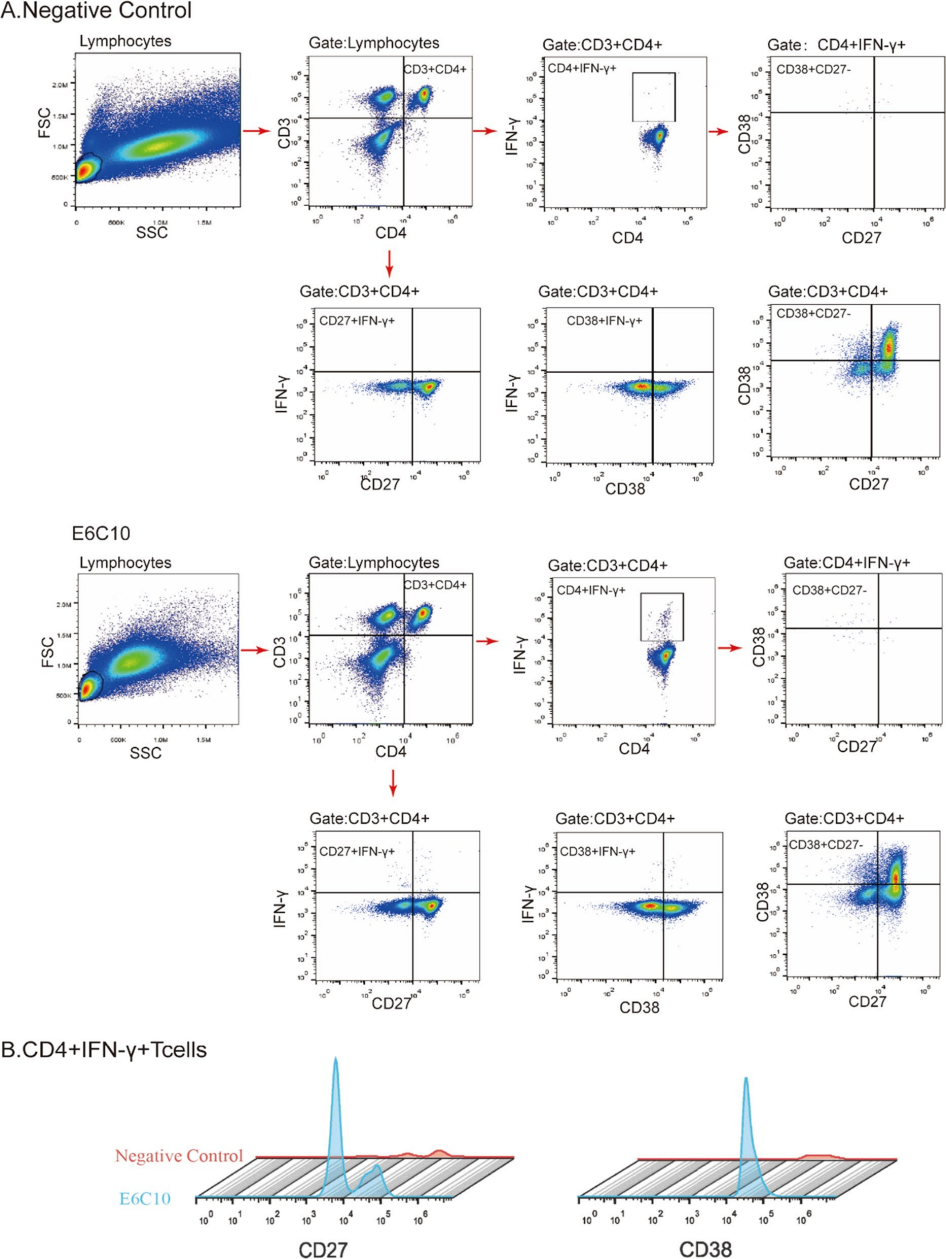
Flow cytometry gating strategy for CD4^+^IFN-γ^+^CD38^+^CD27^−^ T cells from the peripheral blood lymphocyte of the participants. **A** Peripheral blood lymphocyte without stimulation by ESAT-6/CFP-10 peptides (negative control) and with 16-h stimulation by ESAT-6/CFP-10 peptides (E6C10). **B** The comparison of CD27 or CD38 expression in ESAT-6/CFP-10 peptides stimulated CD4^+^IFN-γ^+^ T cells to the unstimulated cells

**Fig. 3:**
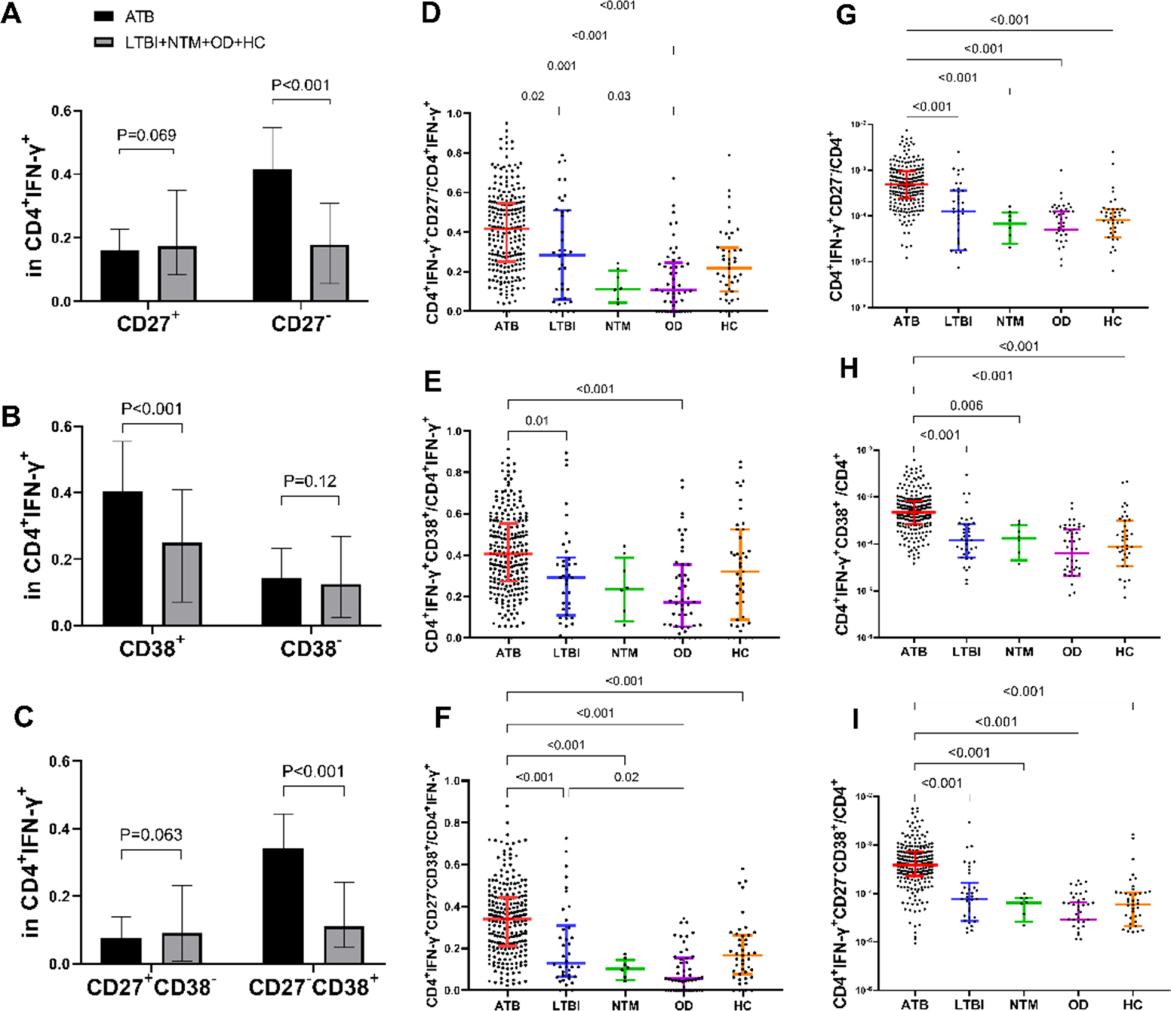
The frequency of CD27 and CD38-expressing populations in CD4^+^ IFN-γ^+^ T cells and in CD4^+^ T cells for the various groups. **A** CD4^+^IFN-γ^+^CD27^+^ and CD4^+^IFN^−^γ^+^CD27^−^, **B** CD4^+^IFN^−^γ^+^CD38^+^ and CD4^+^IFN-γ^+^CD38^−^, **C** CD4^+^IFN^−^γ^+^CD38^+^CD27^−^ and CD4^+^IFN-γ^+^CD27^+^CD38^−^, **D** CD4^+^IFN^−^γ^+^CD27^−^, **E** CD4^+^IFN^−^γ^+^CD38^+^, and **F** CD4^+^IFN^−^γ^+^CD38^+^CD27^−^ in CD4^+^ IFN-γ^+^ T cell in peripheral blood stimulated with ESAT 6/CFP10 were compared in ATB and other groups. The phenotypic markers **G** CD4^+^IFN^−^γ^+^CD27^−^, **H** CD4^+^IFN^−^γ^+^CD38^+^, and **I** CD4^+^IFN^−^γ^+^CD38^+^CD27^−^ on CD4^+^ T cell in peripheral blood stimulated with ESAT 6/CFP10 in each group was compared. *ATB* active tuberculosis, *LTBI* latent tuberculosis infection, *NTM* non-tuberculous mycobacterium pulmonary disease, *OD* other respiratory diseases, *HC* healthy control

The CD27 or CD38 expression in ESAT-6/CFP-10 peptides stimulated CD4^+^IFN-γ^+^ T cells was compared with the unstimulated cells (Fig. 2). The rates of CD27^−^, CD38^+^, and CD27^−^CD38^+^ were significantly higher in the ATB group than in the non-ATB groups (all P < 0.001) (Fig. 3A–C). This also indicates that CD27^−^, CD38^+^, and CD27^−^CD38^+^ are mainly found in the CD4^+^IFN-γ^+^ T cells of ATB patients. The ratio of CD4^+^IFN-γ^+^CD27^−^ and CD4^+^IFN-γ^+^CD38^+^CD27^−^ profiles in CD4^+^IFN-γ^+^ cells stimulated by ESAT 6/CFP10 in the ATB group were higher than that in LTBI (P = 0.02 and P < 0.001, respectively), NTM (P = 0.001 and P < 0.001, respectively), OD (both P < 0.001), and HC groups (both P < 0.001) (Fig. 3D, F). The ratio of CD4^+^IFN-γ^+^CD38^+^ profile in CD4^+^IFN-γ^+^ cells of the ATB group was higher than the LTBI (P = 0.01) and OD groups (all P < 0.001), but no difference was found between the ATB and the NTM or HC group (Fig. 3E). The rates of CD4^+^IFN-γ^+^CD38^+^, CD4^+^IFN-γ^+^CD27^−^, and CD4^+^IFN-γ^+^CD38^+^CD27^−^ profiles in CD4^+^ T cell subsets in the ATB group were significantly higher than in the other groups (all P < 0.001) (Fig. 3G–I).

**Fig. 4:**
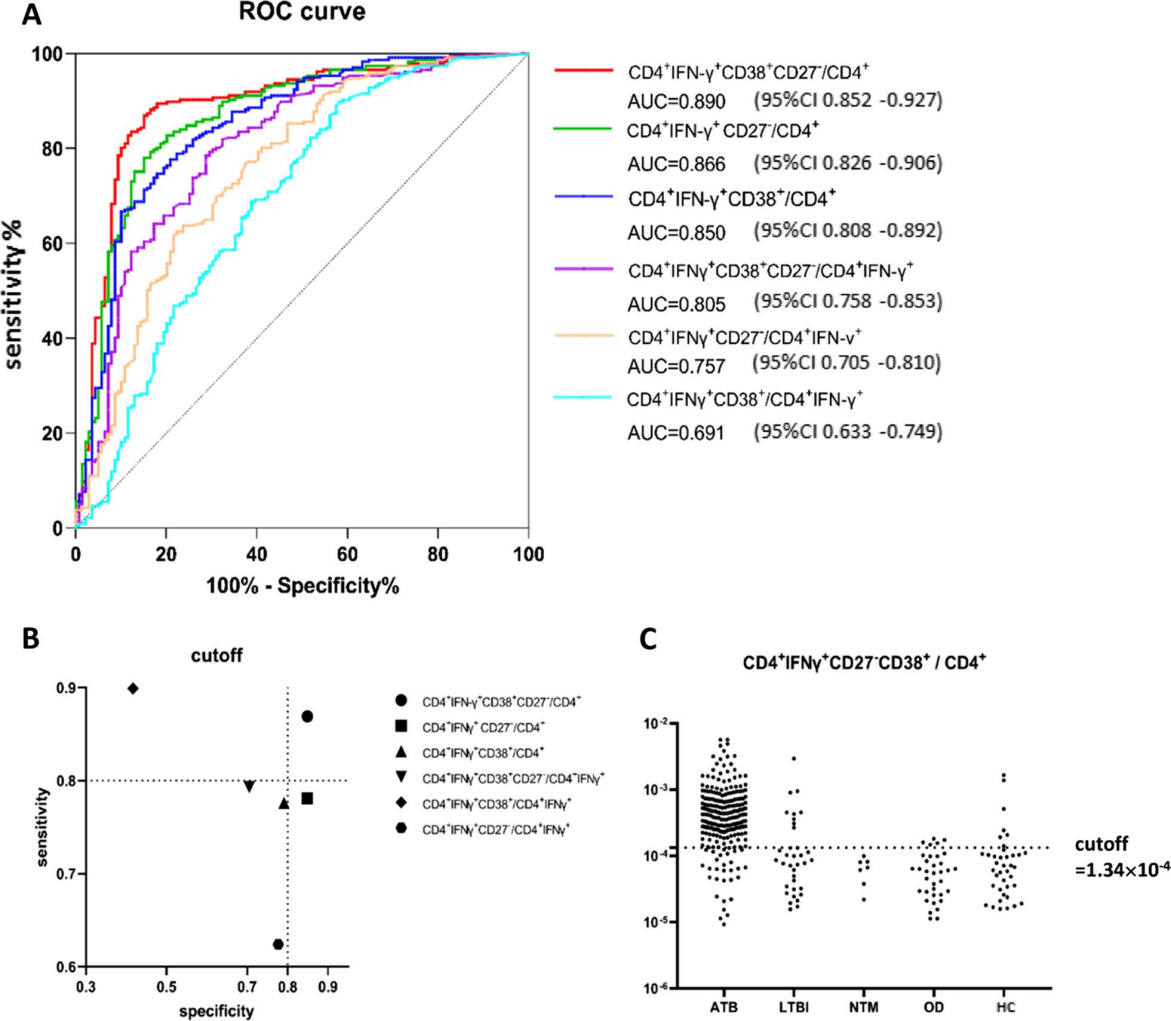
Receiver operating characteristics (ROC) curves and accuracy for the diagnosis of active tuberculosis (ATB). **A** ROC curves of different indicators for the diagnosis of ATB. **B** Sensitivity and specificity corresponding to the cutoff values. The dotted line indicates 80% sensitivity and 80% specificity. **C** The dotted line represents the cutoff value of CD4^+^IFN-γ^+^CD38^+^CD27^−^/CD4^+^ as 1.34 × 10^–4^. *ATB* active tuberculosis, *LTBI* latent tuberculosis infection, *NTM* non-tuberculous mycobacterium pulmonary disease, *OD* other respiratory diseases, *HC* healthy control

The diagnostic values (including sensitivity, specificity, negative predictive value, positive predictive value, positive likelihood ratio, negative likelihood ratio, and accuracy) of CD4^+^IFN-γ^+^CD38^+^/CD4^+^IFN-γ^+^, CD4^+^IFN-γ^+^CD27^−^/CD4^+^IFN-γ^+^, CD4^+^IFN-γ^+^CD38^+^CD27^−^/CD4^+^IFN-γ^+^, CD4^+^IFN-γ^+^CD38^+^CD27^−^/CD4^+^, CD4^+^IFN-γ^+^CD38^+^/CD4^+^, CD4^+^IFN-γ^+^CD27^−^/CD4^+^ for the diagnosis and differential diagnosis of TB were evaluated (Table 2). CD4^+^IFN-γ^+^CD38^+^/CD4^+^IFN-γ^+^ had the highest sensitivity (0.899) and CD4^+^IFN-γ^+^CD38^+^CD27^−^/CD4^+^ had the highest specificity (0.849). ROC curves for ATB diagnosis were performed and the AUCs of the six markers were greater than 0.7 except CD4^+^IFN-γ^+^CD38^+^/CD4^+^ IFN-γ^+^ (Fig. 4A). The AUC of CD4^+^IFN-γ^+^CD38^+^CD27^−^/CD4^+^, which was 0.890, was the highest compared with the other markers (Delong test, P < 0.05), indicating that the CD4^+^IFN-γ^+^CD38^+^CD27^−^/CD4^+^ had the highest value in the diagnosis and differential diagnosis of ATB. At the cutoff value, the sensitivity and specificity of CD4^+^IFN-γ^+^CD38^+^CD27^−^/CD4^+^ were both above 80% (Fig. 4B). At the cutoff value of 1.34 × 10^–4^, CD4^+^IFN-γ^+^CD38^+^CD27^−^/CD4^+^ exhibited good differential diagnosis ability for ATB, as most ATB cases had CD4^+^IFN-γ^+^CD38^+^CD27^−^/CD4^+^ values over 1.34 × 10^–4^ and most non-ATB cases had values below 1.34 × 10^–4^ (Fig. 4C). The sensitivity, specificity, and accuracy of CD4^+^IFNγ^+^CD38^+^CD27^−^/CD4^+^ were 0.869, 0.849, and 0.862, respectively. The positive predictive value was 0.907, and the negative predictive value was 0.792.

**Table 2:** Diagnostic performance of different phenotypic markers

	Cutoff	Se	Sp	PPV	NPV	FPR	FNR	^+^LR	^−^LR	Accuracy	Youden
CD4^+^IFNγ^+^CD27^−^CD38^+^/CD4^+^	1.34 × 10^–4^	0.869	0.849	0.907	0.792	0.151	0.130	5.753	0.154	0.862	0.718
CD4^+^IFNγ^+^CD27^−^/CD4^+^	2.11 × 10^–4^	0.781	0.849	0.898	0.694	0.151	0.219	5.167	0.258	0.806	0.630
CD4^+^IFNγ^+^CD38^+^/CD4^+^	2.56 × 10^–4^	0.776	0.791	0.864	0.675	0.209	0.224	3.721	0.283	0.782	0.568
CD4^+^IFNγ^+^CD27^−^CD38^+^/CD4^+^IFNγ^+^	1.91 × 10^–1^	0.793	0.705	0.821	0.667	0.295	0.207	2.689	0.293	0.761	0.498
CD4^+^IFNγ^+^CD27^−^/CD4^+^IFNγ^+^	3.21 × 10^–1^	0.624	0.777	0.827	0.548	0.223	0.376	2.800	0.483	0.681	0.401
CD4^+^IFNγ^+^CD38^+^/CD4^+^IFNγ^+^	1.80 × 10^–1^	0.899	0.417	0.724	0.707	0.583	0.101	1.542	0.243	0.721	0.316

*Se* sensitivity, *Sp* specificity, *PPV* positive predictive value, *NPV* negative predictive value, *FPR* false positive rate, *FNR* false-negative rate, ^*+*^*LR* positive likelihood ratio, ^*−*^*LR* negative likelihood ratio

**Fig. 5:**
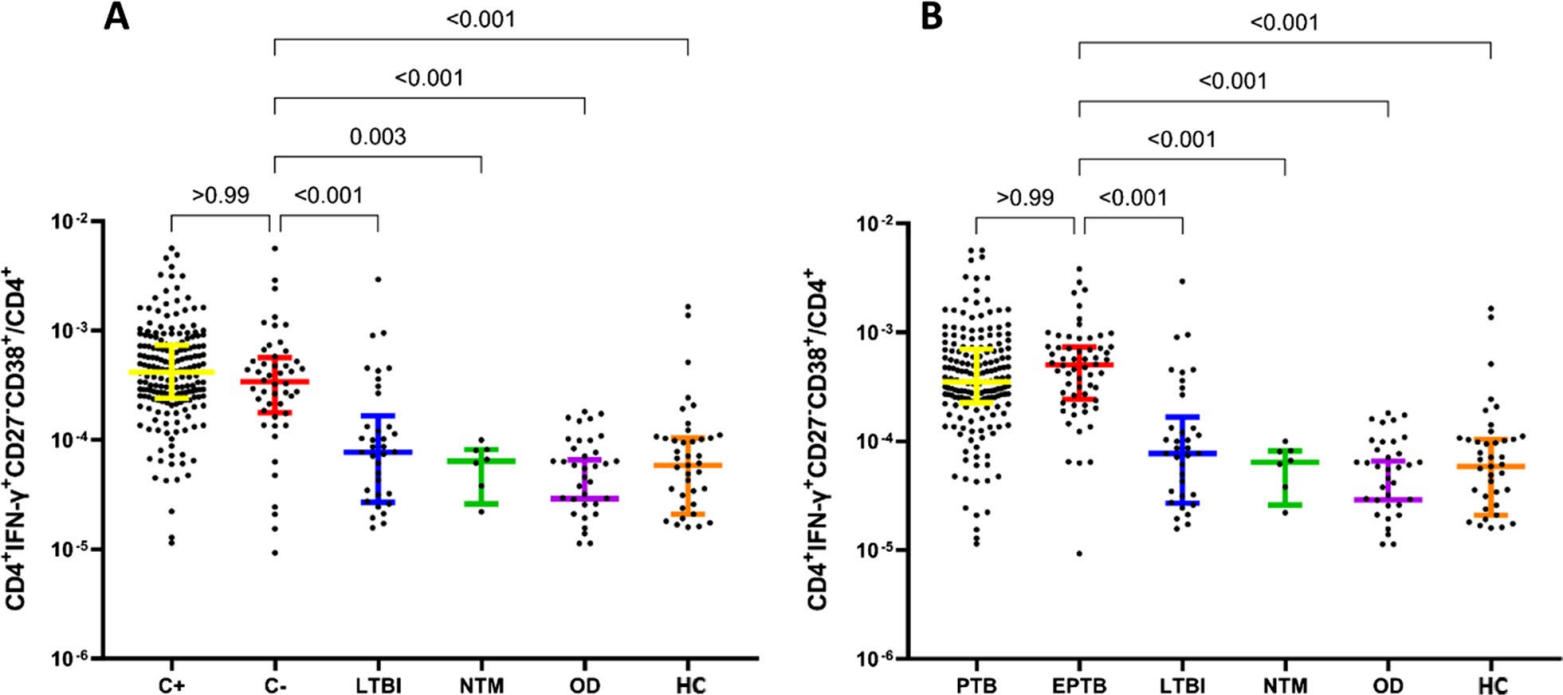
The phenotypic marker CD4^+^IFN^−^γ^+^CD38^+^CD27^−^ on CD4^+^ cells in different subgroups. **A** The phenotypic marker CD4^+^IFN-γ^+^CD38^+^CD27^−^ on CD4^+^ cells with negative TB culture stimulated with ESAT 6/CFP10. **B** The phenotypic marker CD4^+^IFN-γ^+^CD38^+^CD27^−^ on CD4^+^ cells with EPTB stimulated with ESAT 6/CFP10. C^+^: TB culture^+^; C^−^: TB culture^−^; PTB: pulmonary tuberculosis; EPTB: extrapulmonary tuberculosis; LTBI: latent tuberculosis infection; NTM: non-tuberculous mycobacterium pulmonary disease; OD: other respiratory diseases; HC: healthy control

In addition, we investigated the difference in the rates of CD4^+^IFN-γ^+^CD38^+^CD27^−^/CD4^+^ between TB culture-negative (culture^−^) and TB culture-positive (culture^+^) ATB patients. Among the 237 ATB cases, 189 cases were TB culture^+^, and 48 were TB culture^−^. The rates of CD4^+^IFN-γ^+^CD38^+^CD27^−^ in CD4^+^ T cells stimulated by ESAT 6/CFP10 showed no significant difference between TB culture^+^ and TB culture^−^ (P > 0.05). Compared with the other groups, the rates of CD4^+^IFN-γ^+^CD38^+^CD27^−^ in the TB culture^−^ group were higher than in the LTBI (P < 0.001), NTM (P = 0.003), OD (P < 0.001), and HC groups (P < 0.001) (Fig. 5A). The rates of CD4^+^IFN-γ^+^CD38^+^CD27^−^ on CD4^+^ cells of PTB and EPTB were also compared (Fig. 5B). There were 177 cases of PTB and 60 cases of EPTB among the 237 ATB patients. The rates of CD4^+^IFN-γ^+^CD38^+^CD27^−^ in CD4^+^ cells showed no significant difference between PTB and EPTB (P > 0.05). The rates of CD4^+^IFN-γ^+^CD38^+^CD27^−^ in EPTB were higher than in the LTBI, NTM, OD, and HC groups (all P < 0.001). Therefore, CD4^+^IFN-γ^+^CD38^+^CD27^−^ could also be used as a marker the diagnosis and differential diagnosis of ATB patients with TB culture^−^ and EPTB.

## Discussion

The diagnostic performance of CD27^−^CD38^+^IFN-γ^+^CD4^+^/CD4^+^ and other MTB-specific phenotypic markers for ATB diagnosis was evaluated in this study. The AUC of CD4^+^IFN-γ^+^CD38^+^CD27^−^/CD4^+^ for the diagnosis of ATB was the highest (0.890), and the sensitivity and specificity was 0.869 and 0.849 with the optimal cutoff value of CD4^+^IFN-γ^+^CD38^+^CD27^−^/CD4^+^ as 1.34 × 10^–4^. Therefore, CD27^−^CD38^+^IFN-γ^+^CD4^+^/CD4^+^ might be an effective biomarker for ATB diagnosis and differential diagnosis in future clinical application.

In recent years, the research on T cell-related molecular markers has been a hot topic. Xu et al. [27] evaluated CD27 in CD27^−^IFN-γ^+^CD4^+^ T cells for differential diagnosis in TB-unexposed healthy people, TB contacts, and smear-negative TB and concluded that the percentage of CD27^−^IFN-γ^+^CD4^+^ cells can distinguish smear-negative TB patients from the other two groups (AUC = 0.88, sensitivity 82.1%, specificity 80.0%). The study focused on the differential diagnosis of sputum smear-negative ATB and LTB, but many of the smear-negative patients were culture-positive. There are still some ATB patients with both negative smear and culture, and it is challenging to distinguish ATB from LTBI in these patients. Therefore, it is more meaningful to compare ATB with LTBI in sputum culture-negative, which is also one of the key contents of the present study. Latorre et al. [25] found that the rates of CD27^−^ and CCR4^+^ in IFN-γ^+^ TNF-α^+^CD4^+^ T cells stimulated by ESAT^−^6/CFP^−^10 or PPD had a high diagnostic value and a high diagnostic accuracy between ATB and LTBI, but the ATB and LTBI participants in the study were enrolled within the first 4 weeks of initiation of anti-TB therapy or prophylactic anti-TB therapy, and it is questionable whether anti-TB therapy interfered with the results. Silveira-Mattos et al. [26] focused on CD38, HLADR, and Ki67. The results showed that the rates of CD38^+^, HLADR^+^, or Ki67^+^ in IFN-γ^+^CD4^+^ T cells could differentiate LTBI from ATB. HLADR^+^ and Ki67^+^ could identify EPTB and PTB accurately. HIV infection did not affect the ability of these markers to distinguish between ATB and LTBI, EPTB, and PTB. Still, a large proportion of EPTB tends to be combined with PTB, so the comparison is worth considering.

This study focused on the rates of CD27^−^ and CD38^+^ and their co-occurrence. The results showed that after peripheral blood was stimulated by ESAT 6/CFP10, the rates of CD4^+^IFN-γ^+^CD27^−^ and CD4^+^IFN-γ^+^CD27^−^ on CD4^+^ IFN-γ^+^ cells of ATB were higher than in the other groups. The rate of CD4^+^IFN-γ^+^CD38^+^ in ATB was higher than in LTBI and OD, but there were no differences with NTM and HC groups. The rates of CD4^+^IFN-γ^+^CD38^+^, CD4^+^IFN-γ^+^CD27^−^, and CD4^+^IFN-γ^+^CD27^−^CD38^+^ subsets in CD4^+^ cells were higher in ATB than in the other groups. Similar results were observed for the rates of CD4^+^IFN-γ^+^CD38^+^ and CD4^+^IFN^−^γ^+^CD27^−^, consistent with the previous study mentioned above. Nevertheless, there are few studies on CD4^+^IFN-γ^+^CD27^−^CD38^+^, and additional studies are necessary to strengthen the results. ROC curves were performed for the proportions of CD4^+^IFN-γ^+^CD38^+^, CD4^+^IFN-γ^+^CD27^−^, and CD4^+^IFN-γ^+^CD27^−^CD38^+^ in CD4^+^ and CD4^+^ IFN-γ^+^ as diagnostic indexes. According to their AUC, CD4^+^IFN-γ^+^CD27^−^/CD4^+^IFN-γ^+^ and CD4^+^IFN-γ^+^CD27^−^CD38^+^/CD4^+^IFN-γ^+^ have diagnostic value for ATB. The AUC of CD4^+^IFN-γ^+^CD27^−^CD38^+^/CD4^+^ was 0.890, indicating the highest diagnosis value. The reason that the diagnostic value of each biomarker on CD4^+^ cells is higher than CD4^+^IFN-γ^+^ may be related to the both effect of CD27^−^CD38^+^ and IFN-γ, but the exact mechanisms remain to be explored in future research [32].

As it is difficult to diagnose culture-negative TB clinically, acid-fast sputum staining tests cannot distinguish TB from NTM. In countries and regions with relatively poor public health resources, the Xpert MTB/RIF test is not easily accessible. On the other hand, EPTB is more difficult to diagnose than PTB. The diagnosis is usually made by pathology, excluding other diseases and diagnostic treatment, which is expensive in time and resources. In this study, the results showed no difference of CD4^+^IFN-γ^+^CD27^−^CD38^+^ on CD4^+^ neither between TB culture^+^ and TB culture^−^ nor between PTB and EPTB. Still, the rates were higher in TB culture^−^ and EPTB than in the LTBI, NTM, OD, and HC groups. Therefore, CD4^+^IFN-γ^+^CD27^−^CD38^+^ cell subsets could be helpful for culture^−^ TB and EPTB diagnosis.

There are several kinds of MTB antigens used in the stimulation, including MTB-PPD (MTB purified protein derivative). The sensitivity of PPD is usually higher than ESAT6/CFP10 etc. for the in vitro stimulation, but MTB-PPD can also stimulate the T cell response for the individuals who have taken BCG Vaccine, which greatly reduce the specificity of the test, especially in China. ESAT6/CFP10 does not exist in BCG Vaccine, so we used ESAT6/CFP10 as stimulator, and in this study we collected at least 100,000 CD4^+^ cells for each blood sample to ensure the sensitivity of ESAT6/CFP10 alone.

This study has certain limitations. The study aimed to explore the indicators for ATB diagnosis, and the phenotypic markers in the ATB group were compared with the LTBI, NTM, OD, and HC groups because the differential diagnosis of these groups is also of great clinical value. Unfortunately, the sample size for the NTM group was relatively small, mainly because it was a single-center study with a limited sample size. Therefore, multicenter studies with larger samples should be carried out. In addition, this study did not examine the effects of treatments on CD4^+^IFN-γ^+^CD27^−^CD38^+^ cell subsets. The study by Ahmed [29] showed Phenotypic changes of MTB-specific T cells are potential surrogate markers for tuberculosis treatment efficacy and can help to discriminate between aTB [profile: CD38(pos), CD27(low)) and latent MTB infection (CD38(neg), CD27(high)], which is consistent with our results. The study also examined changes in markers after anti-TB treatment, which unfortunately was not studied in our study and will be further analyzed in a follow-up study.

In conclusion, CD27^−^CD38^+^IFN-γ^+^CD4^+^/CD4^+^ might be a potential biomarker for TB diagnosis and differential diagnosis.

## Data Availability

The datasets used and/or analyzed during the current study are available from the corresponding author on reasonable request.
